# What is missing from how we measure and understand the experience of poverty and deprivation in population health analyses?

**DOI:** 10.1093/eurpub/ckad174

**Published:** 2023-10-20

**Authors:** Katharine Timpson, Gerry McCartney, David Walsh, Berengere Chabanis

**Affiliations:** Glasgow Centre for Population Health, Glasgow, UK; School of Social and Political Sciences, University of Glasgow, Glasgow, UK; Glasgow Centre for Population Health, Glasgow, UK; Glasgow Centre for Population Health, Glasgow, UK

## Abstract

**Background:**

Explaining why some populations are healthier than others is a core task of epidemiology. Socioeconomic position (SEP), encompassing a broad range of exposures relating to economic circumstances, social class and deprivation, is an important explanation, but lacks a comprehensive framework for understanding the range of relevant exposures it encompasses.

**Methods:**

We reviewed existing literature on experiential accounts of poverty through database searching and the identification of relevant material by experts. We mapped relevant concepts into a complex systems diagram. We developed this diagram through a process of consultation with academic experts and experts with direct experience of poverty. Finally, we categorized concepts on the basis of whether they have previously been measured, their importance to the causal flow of the diagram, and their importance to those consulted, creating a list of priorities for future measurement.

**Results:**

There are a great many aspects of SEP which are not frequently measured or used in epidemiological research and, for some of these, work is needed to better conceptualize and develop measures. Potentially important missing aspects include stigma, social class processes, access to education, sense of lost potential, neighbourhoods, fairness and justice, emotional labour, masking poverty, being (in)visible, costs, and experiences of power.

**Conclusions:**

Analyses seeking to understand the extent to which SEP exposures explain differences in the health of populations are likely to benefit from a comprehensive understanding of the range and inter-relationships between different aspects of SEP. More research to better conceptualize and measure these aspects is now needed.

## Introduction

An objective of many public health research projects is to explain differences and inequalities^1^ in health trends between and within populations. Most frameworks for understanding these differences, such as ‘fundamental causes’,^2^ and ‘determinants of health’, foreground the importance of socioeconomic exposures such as income, wealth, power, deprivation and poverty as important explanations.^3^^,^^4^ However, a series of studies have identified populations with similar exposure to key measures of ‘socioeconomic position’ (SEP, as a catch-all term for exposure to a wide range of socioeconomic factors^5^), but substantially varying population health outcomes.^6–10^ This has raised the important question of whether the remaining differences between populations are a function of inadequate theorization (i.e. consideration of too narrow a range of SEP mechanisms),^11^ inadequate measurement (which could be due to a lack of measures of important exposures, a lack of data, or data biases)^12^ or due to other exposures.^12^ In the latter case, it has been argued that other exposures may exacerbate the impact of SEP on health, acting as ‘effect modifiers’ or ‘deprivation amplifiers’.^12^^,^^13^ This article, however, focuses on the first two of these explanations—the inadequate theorization and measurement of SEP—the resolution of which is needed for the understanding of whether residual health differences (or inequalities, if systemic and unfair^1^) between populations after accounting for measures of SEP are indeed due, at least in part, to differential SEP exposures.

Existing theories linking SEP to health are either focused on specific exposures or pathways (e.g. power^3^^,^^4^ or social class relationships^11^) or do not sufficiently unpack the range of SEP exposures and their interlinkages for this purpose.^2^,^14–17^ Sometimes SEP measures have been used to capture particular exposures and mechanisms, whilst in other analyses SEP measures are simply used to rank populations in order to measure inequalities or to understand the importance of other non-SEP explanations.^5^^,^^11^ Many studies have used single measures of SEP in isolation, whilst others have attempted to combine measures in recognition of the range of interlinked mechanisms in operation.^18–20^

At the individual level, absolute and relative income,^21^ wealth,^22^ educational attainment,^23^ employment,^24^ car ownership,^25^ receipt of social security benefits^10^ and occupational social class^11^^,^^18^ are all commonly used SEP concepts, and each has a variety of measures. Individuals are nested within households, and some concepts and measures are operationalized at that level in recognition that resources and obligations (including caring duties and the payment of bills) can be shared at that level, albeit this sharing can often be inequitable and gendered.^26^

Individuals and households are in turn nested within communities, both of interest and of place. Area-level measures of SEP are frequently used measures in epidemiological analyses, particularly because they are readily available in many places through the use of spatially linked administrative datasets, and have a low risk of breaching privacy. These are usually termed area deprivation measures and can consist of single measures (e.g. the prevalence in the population of income- or employment-related benefits) or weighted measures of a wide range of spatial information (including reported crime, access to services, health outcomes as well as income- and employment-benefit claims) to create indices of multiple deprivation. Area deprivation measures are used in different ways. As an approximation of individual SEP, they are subject to the ecological fallacy and misclassify a large proportion of the population.^27^ At area level, they can provide a means of understanding exposure at the community level, but often this is simply an average of individual experiences and exposures rather than measures of genuine area-level phenomena.^28^ However, some measures are more explicit in considering the shared exposures (e.g. to the physical environment) or resources (e.g. service availability), albeit few are able to capture differential exposure or access or exposure within areas, complicated further by people moving between areas.^28^ The differential experiences within a shared context have been a recent theme of poverty research: price inflation has been found to be higher for people on lower incomes,^29^ and the costs of living for people living in poverty have been demonstrated to be higher than for the rest of the population.^30^^,^^31^

Rooted in experiential accounts of SEP,^32–34^ and in recognition of the need for, and benefits from, participatory research,^35^ there is a renewed emphasis on conceptualizing and measuring psychological impacts and the impacts of SEP on interpersonal relationships. This includes concepts and experiences such as a sense of identity, self-esteem, control over decisions, stigma and shame,^34^^,^^36^^,^^37^ all of which are structured and nested within broader social and power structures.

Despite this plethora of SEP measures, there remain two central problems. First, there is a broad range of exposures which encompass SEP, including all the dimensions of poverty, deprivation and social class. Indeed, even comprehensive analyses are limited by the range of measures and data available to fully understand the experience of SEP across and between populations.^12^ Second, the inter-relationships between different aspects of SEP are arguably under-theorized, meaning that the causal pathways are not well understood, leading to problems in how research studies of the relationship between aspects of SEP are conceptualized, with the attendant risks of over- and under-adjustment.

This study aims to provide the groundwork for future measurement and comparison of SEP exposure and experience between populations for understanding population health differences. Specifically, we investigate which aspects of the experience of poverty, deprivation, inequality and discrimination are likely to have been insufficiently captured by the types of measures used in previous analyses of poverty and mortality? How can we use this knowledge to better understand, measure and compare experiences of poverty internationally and in the UK?

## Methods

We addressed the research questions by working through five stages:

### Literature review and key informant consultation

We contacted UK experts in the field of poverty and its measurement, and asked them to direct us to key areas of relevant literature. We began by contacting eight researchers whose work we were aware of and who had a focus on the experiences of people living in poverty—the area of measurement which we wanted to develop. These were academic researchers who came from various backgrounds and academic departments, including social policy, policy studies, education, housing, politics, geography, sociology and equality studies. Their work covered a range of topics, including different understandings of the structures that cause poverty, the experiences of people in poverty, how those experiences vary based on personal characteristics (such as gender or age), and the impact of policy responses on poverty. We snowballed our list by asking respondents to nominate others (resulting in 23 experts in total, of whom 17 responded). Respondents suggested specific citations, authors, topics and keywords. Through this, and through the reference lists included within the suggested literature, we identified a total of 169 relevant citations.

### Concept mapping

The citations assessed as relevant were varied in type (including measures, description of concepts, accounts of the causes or impacts of poverty, and personal accounts). To identify areas of commonality and tension across the literature we extracted and tabulated relevant terms. We summarized the concepts from the literature into several groups, which are discussed in detail in Supplementary appendix S1. However, the heterogeneity, overlaps and conflicts across the literature meant that it was difficult to convey the complexity of the literature to experts for feedback with a written summary. This led us to a diagrammatic mapping exercise. Concepts were colour-coded in terms of the extent to which they had been measured in previous relevant research^9^ to identify clusters, or causal pathways, of under-measured concepts. We drew on the methods used for the Foresight obesity diagram, which was designed to create a ‘whole systems’ overview of the multiple, complex, and intertwining causes of obesity.^38^ Likewise, we utilized a broadly ‘dialectical’ approach (our dialectical approach involved discussion amongst the authors, and iteration across concepts and the resulting diagram, in order that the co-dependence, fit and tensions across areas was exposed and then resolved) to highlight the SEP exposures and mechanisms that we identified, and how these were theorized to cause health, illness and mortality in a population. We used Kumu concept mapping software (https://www.kumu.io/) to create the diagram.

### Engagement with academic and literature experts

After initial piloting and editing for clarity, we gathered a group of literature experts (including some of those consulted in phase 1), to establish whether they felt we had summarized the literature accurately, and what was still missing. We edited our summary and the diagram once again in light of their feedback.

### Engagement with people with experience of poverty

**Figure 1:**
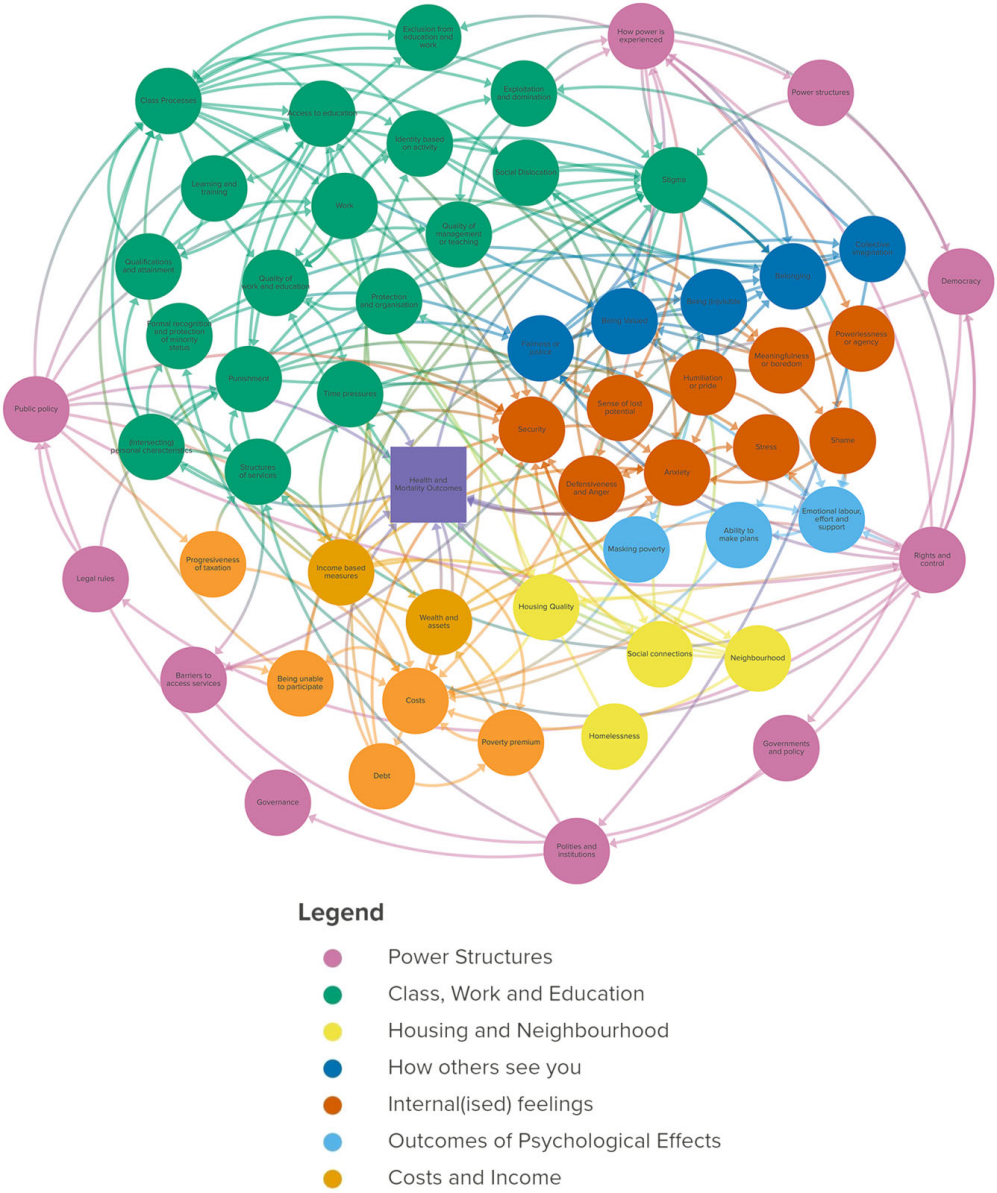
Full diagram.

We then repeated this editing process with a group of experts with experience in poverty. As we had to undertake this online due to COVID-19 restrictions, we approached an existing group of experts. The Poverty Alliance Community Activist Advisory Group (CAAG) (https://www.povertyalliance.org/get-involved/join-our-community-action-group/) is a group of community activists who have experience of poverty and the issues surrounding poverty in Scotland. The group had been meeting online since the start of the pandemic and were thus comfortable with using the required technology. We had two meetings with the group. The first had 20 attendees and the second had 18 attendees (with 15 people present at both meetings). We did not collect demographic data on those attending, because we were not asking them to speak for all people experiencing poverty from the various communities that they were part of, and because we did not want to collect data that might identify individuals and associate them with particular pieces of feedback. Feedback was gathered by breaking the diagram into groups of concepts, presenting each, and then asking for feedback (verbally and using the ‘chat’ function). The feedback allowed us to update the diagram again. By giving tangible examples of how these concepts were connected and how they impacted people’s experiences, the CAAG added to our understanding of many of the concepts and of how they fitted together. We updated the concepts we used, their definitions and their placement and connections within the diagram. This resulted in the final version of the concept diagram shown in figure 1 (discussed in the Results section). The discussions also allowed us to judge which concepts were perceived as more/less important, by highlighting which concepts group members found most relatable, insufficiently understood, or which did not receive enough attention in discussions around poverty.

### Final categorization of concepts

In the final stage of the project, we sought to summarize and categorize the concepts, thereby prioritizing them in relation to future work. The concepts were categorized by the authors in terms of whether or not they had been measured in previous poverty and mortality research; their importance in the context of the structure of the diagram; and their importance to the people we consulted. From these three considerations, we grouped them into four priority groups. These groups do not reflect how important each element is to the overall experience of poverty but instead show priorities for future measurement. As an example, there was no indication from any of the participants that income was an unimportant aspect of poverty. It is given lower priority only because participants and researchers agreed that other concepts are less well understood and measured, and so there is less need to develop new measurements that look at income than, for example, stigma.

## Results

### A complex system map of SEP and health

**Figure 2:**
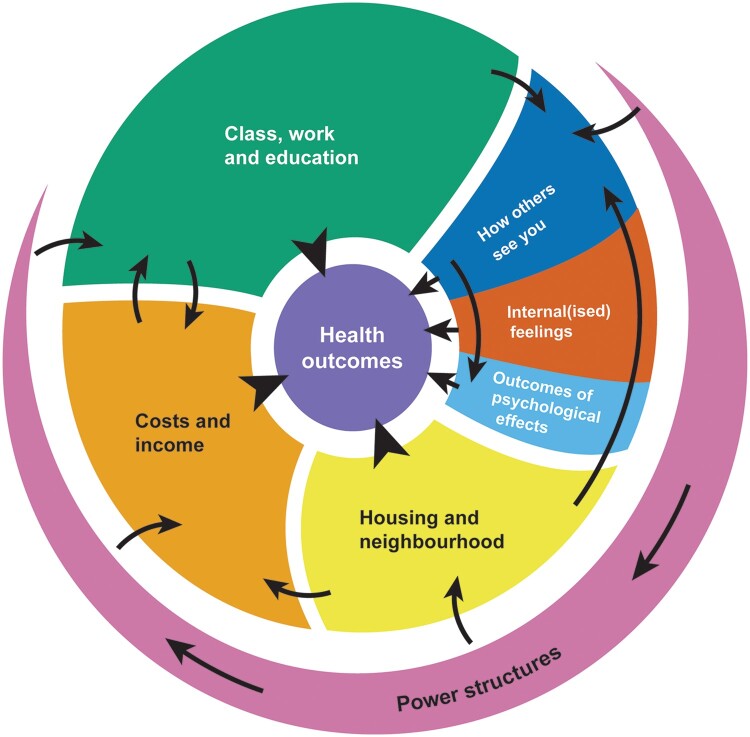
Simplified diagram

Figure 1 shows the final diagram linking the range of SEP concepts into a complex systems diagram, with elements which encompass several concepts noted in table 1 and the full list of concepts and definitions provided in Supplementary appendix S2. Figure 2 is a simplified version of figure 1, summarizing the links between seven concept categories: power structures (purple); housing and neighbourhood (yellow); income and costs (orange); class, work and education (green); how others see you (dark blue); internal(ized) feelings (red); and outcomes of psychological effects (light blue). At the centre of the diagram is ‘health and mortality outcomes’.

**Table 1:** Concepts which contain multiple other concepts

Concept as named in diagram	Concepts included within this
Class processes	Discrimination, habitus and distinction, opportunity hoarding, social closure.
How power is experienced	Being able to see yourself in power, bullying and the misuse of power, having the ability to challenge those in power, membership, political agency and voice, power distribution, varieties of participation.
Housing quality	Tenure type, affordability, size/overcrowding, damp, warmth (and cost and type of fuel), security, furniture, internet access, decor, the amount that you need to move house, suitability, choice of area to live in.
Income-based measures	60% median income, depth of poverty, income distribution, intra-household distributions of wealth and income, periodicity of poverty.
Intersecting personal characteristics	Protected characteristics, not protected characteristics—such as care experience or citizenship status.
Neighbourhood	Accessibility of amenities, physical environment, remoteness, transport.
Power structures	Cultures, ecology, history, relationships between societies (e.g. imperialism).
Social connections	Family, friends/community and household composition.

Figure 1 summarizes what was found in the literature and what was discussed by literature experts and by the CAAG. The diagram is intentionally complex, to reflect the complex ways in which poverty operates and is understood. Figure 2 offers a simplification looking only at the connections between the broad concept groups.

The more structural concepts, such as power and class systems, are on the outside of the diagram, with the concepts that people experience more personally closer to the centre. However, there are many pathways from each concept to the central outcome of worsened health. For example, public policy, located within ‘power structures’, can impact health quite directly, by impacting upon the structure of (health) services. There are also much less direct pathways—working through class systems to influence people’s access to education and work, and in terms of people’s sense of identity based on the work that they can do (or cannot do). Whether this allows people to feel that they belong within a community can provoke internal feelings such as shame and anxiety, impacting mental and physical health. Alternatively, public policy can impact the quality of housing available to people. The higher costs of having to maintain poor-quality housing can mean incurring debt, which can result in stigma. This can lead to trying to mask poverty—a process which takes time, money and effort which people can then not spend on maintaining their health.

Supplementary appendix S1 has more detail on the connections made between concepts and groups. In both the diagram and the summary, some elements are covered in more detail than others—this does not denote greater importance to the overall experience of poverty, but only that there was more focus on developing these areas for future measurement of poverty.

### Prioritization of SEP concepts for future measurement

Prioritization of concepts generated four graded levels for future measurement work (table 2). The highest priority group (including 14 concepts) was identified as the most important to the overall diagram and theorized causal pathways because they had the least redundancy and might reasonably therefore be expected to capture aspects of SEP that would otherwise be missed. In addition, they were described by both the literature experts and the CAAG as being of most importance. The next highest priority group (12 concepts) was similarly ranked by our team in terms of importance to the diagram but was accorded slightly lower priority by the experts. The third group (17 concepts) comprised concepts that either were low priority within expert discussions or were considered low priority on the basis of redundancy within the diagram. For example, defensiveness and anger are distinct from other emotions covered in the diagram; however, participants did not engage with those emotions as much as they did with others such as shame or anxiety. As another example, participants mentioned the importance of being able to see oneself in a position of power; however, within the diagram, this was already incorporated within the larger concept of ‘experiences of power’. The fourth group (five concepts) were not prioritized by either participants or by the team (on the basis of redundancy). For example, security was not perceived as a key topic for the experts, and it was felt that measuring related concepts such as income or housing might adequately capture aspects of security.

**Table 2:** Priorities for future measurement

Group 1: High priority for participants; necessary witdin diagram	
Access to education	Class, work and education
Sense of lost potential	Internal(ized) feelings
Masking poverty	Outcomes of psychological effects
Stigma	Class, work and education
Class processes	Class, work and education
Neighbourhood	Housing and neighbourhood
Fairness or justice	How others see you
Emotional labour, effort and support	Outcomes of psychological effects
Being (in)visible	How others see you
Costs	Costs and income
How power is experienced	Power structures
Anxiety	Internal(ized) feelings
Housing quality	Housing and neighbourhood
Qualifications and attainment	Class, work and education
Group 2: Mid priority for participants; necessary within diagram	
Structures of services	Power structures
Being Valued	How others see you
Collective imagination	How others see you
Exploitation and domination	Class, work and education
Shame	Internal(ized) feelings
Humiliation or pride	Internal(ized) feelings
Debt	Costs and income
Barriers to access services	Power structures
Income-based measures	Costs and income
Social connections	Housing and neighbourhood
Wealth and assets	Costs and income
Time pressures	Class, work and education
Group 3: Low priority for participants; *or* not necessary within the diagram	
Identity based on activity	Class, work and education
Defensiveness and Anger	Internal(ized) feelings
Ability to make plans	Outcomes of psychological effects
Punishment	Class, work and education
Belonging	How others see you
Stress	Internal(ized) feelings
Social dislocation	Class, work and education
Transport	Housing and neighbourhood
Rights and control	Power structures
Being unable to participate	Costs and income
Poverty premium	Costs and income
Quality of management or teaching	Class, work and education
Quality of work and education	Class, work and education
Homelessness	Housing and neighbourhood
Meaningfulness or boredom	Internal(ized) feelings
Work	Class, work and education
Powerlessness or agency	Internal(ized) feelings
Group 4: Low priority for participants; *and* not necessary within diagram	
Security	Internal(ized) feelings
Exclusion from education and work	Class, work and education
Learning and training	Class, work and education
Formal recognition and protection of minority status	Power structures
Protection and organization	Class, work and education
Other concepts: Structural concepts, not to comparatively measure	
Democracy	Power structures
Governance	Power structures
Government and policy	Power structures
(Intersecting) personal characteristics	Class, work and education
Legal rules	Power structures
Polities and institutions	Power structures
Power structures	Power structures
Progressiveness of taxation	Power structures
Public policy	Power structures

## Discussion

### Summary of main findings

There are many different SEP concepts and these have complex relationships with each other and with health outcomes. We have constructed a complex systems diagram, which theorizes how these concepts link together. In doing so, we have identified priority concepts for measurement on the basis of expert opinion and criticality (i.e. lack of redundancy within the complex system diagram). Prioritized concepts for future measurement include stigma, social class processes, access to education, sense of lost potential, neighbourhoods, fairness and justice, emotional labour, masking poverty, being (in)visible, costs and experiences of power.

### Strengths and weaknesses

The main strength of this approach was in consulting people with experience of poverty—both direct personal experience and experience of working with people in poverty. Seeing how the perspectives from this group aligned with, and diverged from, those found in the literature enabled an understanding of how varied the experiences of poverty are, and how much is missed by measurement. Their input focused the process on different concepts and ideas than would otherwise have been prioritized, and their insights guided the results in terms of what should be measured, and in what manner. Another strength was the iterative nature of the project: each stage was built on previous work, with extensive time taken to discuss and integrate each new perspective into the diagram. This work is also rooted in a real-world public health problem—the higher mortality in Glasgow and Scotland even when compared with equally deprived populations, or where SEP differences between populations are adjusted for.^12^ This builds on and extends approaches like those taken by Bray *et al*.,^35^ where participants designed poverty measurements across geographical settings. Speaking to participants from Glasgow and Scotland about their experiences of poverty in those places, where we know we are not fully measuring the impact of poverty,^12^ allowed us to focus on the elements that people considered most important in those contexts. Finally, this work identified, and prioritized, important aspects of life circumstances that have not been previously measured in analyses of health outcomes. This provides a platform for future work in this area.

There are limitations in our approach. The literature review was not exhaustive nor systematic, and thus relevant studies may have been missed. We tried to mitigate this by consulting academic experts. However, there may be relevant areas of the literature that we did not consider. For example, based on the feedback from CAAG, we could have consulted literature experts on discrimination. This is why we would recommend that any future work would begin by undertaking a more thorough literature review focused on one concept or area. There was complexity and conflict within even just the literature consulted for this exercise, and the diagram does not capture all of that, as it attempted instead to summarize the breadth of the literature for participants. Any future work, which would be focused on one area or one concept, would need to engage more fully with the complexities around that area or concept as a first step. In creating the diagram, we simplified some concept areas such as ‘housing’, whilst keeping others, such as ‘internal(ized) emotions’, more complex. This facilitated the presentation of the concepts to expert groups, placing the focus on the areas that we knew needed the most discussion. However, it is possible that in doing this, we might have missed out on more detailed expert feedback in some of those areas which were presented in a simplified way. There have also been criticisms of the use of so-called ‘lived experience’ perspectives in research, as they can be exploitative, extracting accounts from participants which are emotionally draining to give, whilst maintaining the hierarchical relationship between researchers and participants.^39^^,^^40^ Working with an already established group, who are consulted regularly on such issues and who were reimbursed for their input, may have mitigated some of these concerns. Other limitations were identified by the CAAG members, but these are not restricted to this study. First, there is the risk that measuring and identifying populations in poverty can exacerbate stigmatization of areas and social groups.^33^ Second, concern was expressed that emphasizing the emotional impacts of poverty could distract us from looking at the power structures that cause it, linked to evidence that approaches led by ‘lived experience’ groups can result in a less structural or political account of poverty.^39^^,^^40^ Third, concern was expressed that the group could not speak on behalf of all people experiencing poverty, in recognition that experiences are highly heterogenous. This emphasizes the limited generalizability of these findings to the context of Scotland.

### Implications

The principal implication of this work is to provide a focus, and means, for future research to better understand differences in the experience of poverty between populations, not least between the populations of Glasgow, Liverpool and Manchester, and Scotland and England, which was the underlying motivation for this work.^9^^,^^10^^,^^12^ The next steps in this regard are to identify or develop measures for each concept and build these into comparative population surveys to better understand the higher mortality in Glasgow and Scotland after accounting for age, sex and (currently measured) SEP differences.^9^^,^^10^^,^^12^ More generally, researchers seeking to understand population health differences might also wish to consider utilizing this wider group of SEP measures, in line with previous recommendations.^5^^,^^17^ For policymakers, a deeper understanding of how SEP operates in societies, and how this leads to population health and health inequality outcomes, can help to inform priorities and policy development.

## Conclusions

Analyses seeking to understand the extent to which SEP exposures explain differences in the health of populations are likely to benefit from a comprehensive understanding of the range and inter-relationship between different aspects of SEP. More research to better conceptualize and measure concepts that are not routinely included in SEP analyses, including stigma, social class processes, access to education, sense of lost potential, neighbourhoods, fairness and justice, emotional labour, masking poverty, being (in)visible costs, and experiences of power, is now needed.

## Supplementary Material

ckad174_Supplementary_Data

## Data Availability

The data used in this work came from discussions with groups and individuals and, in order to protect their privacy, it cannot be shared publicly.
