# Supplementary material for: What is missing from how we measure and understand the experience of poverty and deprivation in population health analyses?
Source: Eur J Public Health. 2023 Oct 20;33(6):974–80. doi: 10.1093/eurpub/ckad174 (PMC10710332; doi:10.1093/eurpub/ckad174)
Supplement: ckad174_Supplementary_Data [file ckad174_supplementary_data.zip › ckad174_Supplementary_Data/ejph-2023-04-om-0206-File006.docx]

**Appendix 1 – Literature Summary**

**Background**

Conceptual understandings of poverty, and its impact on other outcomes such as health, are complex and detailed, with new work continually emerging that adds further nuance. How poverty is measured has also become more complex over time. However, there remains a gap between what is conceptualised and what is routinely captured by measurement – and so what is acted upon by decision-makers. There is a further gap between conceptualisations and the experiences of people living with poverty. The work reported in this paper sought to identify key experiences and concepts which could be developed into measurements to address these gaps. This involved summarising the measures and concepts, and then taking these summaries to expert groups for comment, additions and changes.

This appendix details the summary that was used to begin those conversations. The summary was undertaken not to determine or detail all of the ways that poverty impacts upon health, or even to encompass every measurement described in the literature. It was to give experts an overview of current measurements and conceptualisations, so that they could help identify what was missing. This summary is included here to detail what areas of the literature were included and how these were grouped and formed the basis of those conversations – which are described further in the main paper.

**Current understandings and measurements of poverty**

Peter Townsend famously defined poverty and deprivation in these terms: “people are relatively deprived if they cannot obtain, at all or sufficiently, the conditions of life - that is, the diets, amenities, standards and services - which allow them to play the roles, participate in the relationships and follow the customary behaviour which is expected of them by virtue of their membership of society. If they lack or are denied resources to obtain access to these conditions of life and so fulfil membership of society, they may be said to be in poverty” (1, p.36). The measures most commonly used in epidemiological research arguably fall well short of Townsend’s description.

*Income*

The currently most commonly used measures primarily consider income levels, using them to determine a threshold within a population, below which people are categorised as living in poverty. The threshold can be a relative (e.g. 60% of median income) or an absolute (e.g. a calculated living cost line) phenomenon, reflecting whether poverty is conceptualised as the ability to fully participate in society and share similar experiences or whether it is defined instead as the ability to ‘get by’ and afford essentials. In many senses both are actually relative phenomena because any attempt to define what is essential is closely informed by societal norms (2). Other commonly used individual measures of poverty include: occupation-based social class (there is a broad range and long history of such measures (3)); educational attainment (4); wealth, inequality (5) and family affluence (6); housing tenure (7); car ownership (as a proxy for income) (8); employment status (9); and receipt of social security payments (10).

Income-based measurement remains essential, if not sufficient, to understanding poverty and should not be lost in trying to find the perfect measure (11), but there are nuances between different ways in which income can be measured. Measures can focus on extremes, such as destitution (12), the persistence or depth of low income (e.g., 13, 14), income inequality (15), financial vulnerability (16), or the various ways that income is shared and allocated within households (17-19) , including the potential for economic abuse (20). The assets, wealth, liabilities and debts attributable to individuals and households also provides information about their ability to participate in societal norms and some measures have measured wealth alongside poverty (e.g., 21, 22).

Poverty may be estimated using a single measure, but other approaches work with indices of multiple deprivation – taking various elements of the experience of poverty at once and weighing them to produce a single figure (23). This has the advantage of giving a comparable estimate for poverty in an area, but often works best over larger scales and is less useful at the local level (24). Indices are also developed for specific aspects, such as fuel poverty, which again use already existing data, amalgamated to estimate experiences of poverty (25).

The work of the Oxford Poverty & Human Development Initiative (26, 27), draws on routinely available administrative datasets, frequently derived from the postcodes of people claiming particular social security benefits, to create reasonably contemporaneous estimates of the prevalence of deprivation. These type of deprivation indices have been developed and used across the UK (e.g., 28, 29). They have the advantages of being cheap to produce and maintain (as they rely on administrative data), can generate estimates for small spatial areas (as they do not rely on survey sampling), and encompass a wide range of relevant data. However, indices based on administrative datasets are limited by the data that is routinely collected and comparable across areas, which was often not initially designed to capture poverty and deprivation, and so they provide only a limited picture of the experience (30).

*Costs*

In order to fully capture poverty, as is explicit in the Townsend definition provided above, the costs of participation in society also need to be considered. This can be incorporated through regular revision of absolute poverty lines to adjust for changing costs. However, at times of stagnating mean incomes and high inflation, more people will experience a change in circumstances even though this could be normalised in relative poverty definitions. Differential inflation also needs consideration (31).

Deprivation studies look directly at the items that people have the resources to access and have made comparisons across the UK (e.g., 32, 33-38). These studies are based on first obtaining a consensus over which items are deemed to be ‘necessities’ and then using those lists to produce surveys asking people whether they have had to go without those necessities because of a lack of money. Changing the list of items means that this sort of study can target various levels, focusing on what level of income is required for basic survival or for full participation in society (39-41).

Hidden costs of participation in society – such as the costs of education (e.g., 42, 43), of pregnancy and having children (44), or of transport (45), or of healthy eating (46), or of managing long term health conditions (47), or of setting up a home (48) – have also been considered by researchers. Each of these can be further heightened by the ‘poverty premium’: the way that certain costs increase for those who have least money (49).

The Social Metrics Commission was set up to establish a consensus around how to measure poverty. It combines several elements of the measures described above. it measures income, assets and necessary costs (such as housing and debt) to calculate available income and then compares that to the costs of necessities. It also considers the depth and persistence of poverty. However, this is calculated at a regional level – so cannot be used to compare, for example, cities (50, 51). The commission also reports on nine sets of lived experience indicators, including social isolation, strained family relationships, labour market access, lack of digital skills and confidence and history of trauma. Measurements are taken to compare those living in poverty with those not living in poverty – showing whether these sets of experiences are more common for people in poverty, as defined by the income, assets and costs measurement. The lived experience aspects are not to measure the extent of poverty, or to look at the differential impacts of poverty on different groups or in different places.

*Experiences of work, education, housing, community and neighbourhoods*

It is also possible, as with health, to measure poverty by self-report. However, this is usually done alongside other measures (e.g., 52), in part because the stigma around poverty is so great that people often reject the labels of being poor or experiencing poverty (53). Research has highlighted several aspects which are key to understanding the experience of poverty, including: work, education, housing, communities and neighbourhoods (54, 55).

Poverty can be an outcome of work, because of how work links to income, but the power structures that determine work conditions and cultures are also influenced by poverty and inequality. Poverty impacts upon the type of work that someone can access, and the conditions attached to it. Good quality work, which is well-paid and has room for progression, can improve self-esteem and happiness, and bad quality work, or a lack of work, or overwork, can be detrimental to health (56, 57). How paid work is balanced with unpaid work, care and non-work is also important to people’s sense of identity and wellbeing, and experiences can differ by class and by income level (56, 58).

Education is also filled with power dynamics, issues of time and control and the need for a sense of meaning and self-esteem. Education is an important part of what is measured as children’s wellbeing and as part of poverty (59, 60) and it also plays into class and classification. It affects children’s futures (61), and can help them overcome childhood experiences of poverty (62), but, more often, education privileges middle class values or knowledge (63, 64) or ignores the needs of poorer children (42), feeding into the class distinctions that shape living in poverty.

The housing and neighbourhoods in which people live are also impacted by and impact upon poverty. Living in poverty often means having limited access to good quality housing and local resources (55, 65). Health can be harmed by physical conditions such as dampness, as well as the security of housing tenure and the length of time spent in poor quality housing (65-67). Homelessness, often causing and caused by poverty, has a detrimental impact on health (68).

Poverty line approaches are criticised for ignoring neighbourhoods, and so having “no reference to the issues of inequality and to power relations in the community within which the poor live” (69, p.55). Neighbourhoods have physical, quantifiable characteristics that can impact health – accessibility of shops, amount of green space, safety – but they are also social spaces. Community spaces and resources can be a ‘lifeline’ and their loss can be harmful (49).

Poverty can cause close relations to be tense or to break down (57, 70, 71). As with work, education, housing and neighbourhood, class processes influence the expectations that people place on relationships – and, therefore, the support that they gain from those relationships (72).

*Psychological strains*

Poverty can also be defined by the psychological strains that it causes (73). Living with poverty feels insecure (65), which prompts a range of emotional responses, from anxiety (74) or fear (75, 76) to depression (77) and despair (78), boredom or a sense of meaninglessness (79) – all of which involve or invoke stress (16). Each of these emotions is discussed in the poverty literature, but the largest focus is on feelings of shame and experiences of stigma. Shame and stigma are difficult to untangle, and are often used interchangeably (73). They are elements in the same process and both crucial to understanding poverty.

Wilkinson and Pickett describe shame as “the range of emotions to do with feeling foolish, stupid, ridiculous, inadequate, defective, incompetent, awkward, exposed, vulnerable and insecure” (80, p.41). For Lister, poverty is “experienced as a shameful and corrosive social relation” (81, p.141). For Sen, it is “the irreducible absolutist core” of poverty (82, p.159) – a constant in the lives of those who cannot afford necessities, and Walker adds that it is also “a structural element in the production of poverty” (73, p.182). Shame alerts us to the judgements of others, which we know have consequences for our social bonds and the identities that we are able to hold (83). Since human beings are social, “we know ourselves partly through the eyes of others” (84, p.52), this also impacts our sense of our own identity. Because of this, “shame figures in most social interactions because although members may only occasionally feel shame, they are constantly anticipating it.” (85, p.97).

Stigma is the social process by which “stereotypes are generated through social distance” (63, p.1019). Disgust, judgement and contempt play roles in allowing more powerful groups to distance themselves, psychologically and often physically or geographically, from those with less power. This results in lowered empathy and heightened social tensions (86). Being the object of stigma means being, and feeling, constantly judged by others whose views are seen as more legitimate than your own, “a very uncomfortable position to occupy.” (87, p.131). Stigma harms people’s relationships with friends and family (88), the wider public, and with institutions and support services (89, 90).

Stigmas are based around disparaging narratives, which are created and fuelled by political decisions and media discussions (86, 91-93). The stigmatising narrative around poverty is that those experiencing it are a societal burden and “lack the ability to create value” (94, p. 194), which is particularly heightened in a neoliberal society (95) where money is so strongly tied to worth: “In a society in which status is increasingly defined by income, it is hard to imagine a more powerful way of telling a large swathe of the population that they are almost worthless than to pay them a quarter of 1 per cent of what someone else in the same company is paid” (96, p.25).

At times, stigma and shame are embraced by policy, used to deter people from actions that policy-makers deem to have detrimental social impacts – such as the term ‘benefits scroungers’ being used to deter people from claiming benefits (73, p.63), or public health campaigns seeking to ‘de-normalise’ smoking (97). However, even when used in this way, they still cause harm to those shamed and stigmatised (73, 80, 86). Shame and stigma are also attached to other characteristics and circumstances. Some of these are things which individuals have no choice over (e.g., age 98, ill-health 99); others are things which do not, in themselves, have negative social impacts (e.g., ethnicity 100). In both cases, the shame and stigma experienced causes harm with no positive outcome. For people who experience multiple power imbalances at once, the effects of shame and stigma can be compounded. All of this impacts on health, because our “bodies are the physical sites where the relations of class, gender, race, sexuality and age come together and are embodied and practised.” (101, p.82).

*Coping with poverty*

Some literature also highlights what people do to deal with the material and emotional impacts of poverty – and what those actions can cost them in terms of their health and wellbeing (102)). Borrowing or prioritising one payment over another may alleviate the experience of deprivation in the immediate term but may increase vulnerability in the future (16). Drawing on the support of friends and family can put stress on those relationships (103). Resilience means taking on stress, which brings ‘invisible’ mental health costs (104). Taking on debt can compound the money worries that come with being on a low income, particularly for those who do not have access to cheap credit (68, 105). Debt recovery practices can be harassing, worsening stress and worry (106). Debt can be complex to measure, as not all debt is formal, but attempts have been made using the Families and Children Survey (106), and the Family Resources Survey (107).

Coping with the shame and stigma that come with poverty pushes people to conceal poverty, or to try and adopt middle class practices – which are expensive (87, p.126, 108). Fulfilling social obligations, to reduce the shame of poverty, can mean going without necessities (30). Pushing against stigma by aligning yourself with more socially acceptable roles, or by emphasising distance from those even worse off creates deeper stigma and resentment (65, 78). This co-construction of shame threatens social bonds and communities (109).

Structural critiques can shift blame away from the self, defending against negative self-perceptions (110), although this can also lead to a lack of agency for individuals, and corresponding apathy (75). Being part of a community, with shared understandings of society and identities, can help people to feel connected (95), but communities are often defined by boundaries – which shut others out or constrain the identities of insiders (111).

These contexts are all influenced by wider societal structures, and many accounts of poverty consider those structures directly, looking at how power is held and maintained in a society, and what impact that has on those living within that society. History is important, since the “social space we occupy has been historically generated” (87, p.128), and a loss of history is experienced as a loss of identity, or potential identity (74).

The quality, availability and cost of a wide range of services (the so-called ‘material’ aspects of the ‘Foundational Economy’ (112)), sometimes characterised as the welfare state type (113), has a direct impact on experiences of poverty and deprivation across local and national political jurisdictions, and has led to calls for the introduction of ‘Universal Basic Services’ to reduce the costs of societal participation (e.g., 114).

*Lived experience accounts*

A recent strand of research has emphasised the need to involve individuals with ‘lived experience’ of poverty in better understanding, and measuring, the related concepts. The voices of those in poverty have been described as ‘quite muted’ and seen as less reliable than ‘objective’ measures (73, p.31). For example, given a lack of meaningful participation in decision-making is itself an aspect of poverty (115), Bray et al (116) argued that the way that poverty is measured must itself be participatory, and that those who experience it are best able to decide how to measure it. They therefore used a deliberative participation method of co-production to produce a diagram encapsulating the experience of poverty. Spanning six countries, the project included participants from groups of people experiencing poverty, as well as academics studying poverty and social welfare workers who see the effects of poverty, with priority given to marginalised groups. This work delineated three core dimensions, three relational dimensions and three dimensions referred to as ‘privations’ of poverty. It noted that measurement generally focused on the last group, made up of: material and social deprivation, insufficient and insecure income and lack of decent work. This study emphasised that the core experiences and the relationship aspects were at least as important to how poverty felt (116). The UK section of the project focused on six dimensions, looking at disempowering structures, financial insecurity, damaged health, stigma, lack of control over choices and the unrecognised contributions of people living in poverty (117). Participants felt that the disempowering structures, systems and policies were the most ‘intolerable’ dimension and the ones which needed to be tackled first. The report ended with a set of messages for work and policy, including that “there is a need for better indicators of poverty that emphasise and capture the human experience of poverty” (117, p.38). This work has fed into the lived experience measures of the Social Metrics Commission, noted above (50, 51).

**Reference List**

1. Townsend P. The international analysis of poverty. London: Harvester Wheatsheaf; 1993.

2. Popham F. Deprivation is a relative concept? Absolutely! Journal of Epidemiology and Community Health. 2015;69(3):199-200.

3. Walsh D. An analysis of the extent to which socio-economic deprivation explains higher mortality in Glasgow in comparison with other post-industrial UK cities, and an investigation of other possible explanations: University of Glasgow 2014.

4. Khalatbari-Soltani S., Maccora J., Blyth F.M., Joannès C., M. K-I. Measuring education in the context of health inequalities. International Journal of Epidemiology. 2022;51(3):701-8.

5. J. H, M. B, S. J. An anatomy of economic inequality in the UK: Report of the National Equality Panel. London: Government Equalities Office; 2010

6. Currie C. LKTJ. Health Behaviour in School-aged Children: World Health Organization Collaborative Cross-National Study (HBSC): findings from the 2006 HBSC survey in Scotland. Edinburgh: University of Edinburgh; 2008.

7. Ellaway A., Macdonald L, A. K. Are housing tenure and car access still associated with health? A repeat cross-sectional study of UK adults over a 13-year period. British Medical Journal Open. 2016;6:e01226.

8. G. S, D. W, G. M. Is ‘excess’ mortality in Glasgow an artefact of measurement? Public Health. 2015;129(6):684-90.

9. Fone D., Dunstan F., Williams G., Lloyd K., S. P. Places, people and mental health: A multilevel analysis of economic inactivity. Social Science & Medicine. 2007;64(3):633-45.

10. Schofield L. WD, Munoz-Arroyo R., G. M. Dying younger in Scotland: trends in mortality and deprivation relative to England and Wales, 1981-2011. Health & Place, . 2016;40:106-15.

11. Child Poverty Action Group. Ending Child Poverty by 2020: Progress Made and Lessons Learned. London: Child Poverty Action Group; 2012.

12. Fitzpatrick S, Bramley G, Sosenko F, Blenkinsopp J, Wood J, Johnsen S, et al. Destitution in the UK 2018. York: Joseph Rowantree Foundation; 2018.

13. Culliney M, Haux T, McKay S. Family Structure and Poverty. Joseph Rowntree Foundation; 2013.

14. Government S. Severe Poverty in Scotland Communities Analytical Service: Scottish Government; 2015 [Available from: <https://www.gov.scot/publications/severe-poverty-scotland/>

15. Wilkinson RG, Pickett KE. Income inequality and population health: a review and explanation of the evidence. Social Science and Medicine. 2006;62(7):1768-84.

16. Treanor M. The Effects of Financial Vulnerability and Mothers’ Emotional Distress on Child Social, Emotional and Behavioural Well-Being: A Structural Equation Model. Sociology. 2016;50(4):673-94.

17. Bennett F. Researching Within-Household Distribution: Overview, Developments, Debates, and Methodological Challenges. Journal of Marriage and Family. 2013;75(3):582-97.

18. Bennett F, Daly M. Poverty Through a Gender Lens: Evidence and Policy Review on Gender and Poverty. Oxford: University of Oxford; 2014.

19. Daly M. Towards a theorization of the relationship between poverty and family. Social Policy Admin. 2018;52:565-77.

20. Adams AE, Sullivan CM, Bybee D, MR. G. Development of the Scale of Economic Abuse. Violence Against Women 2008. 2008;14(5):563-88.

21. Dorling D, Rigby J, Wheeler B, Ballas D, Thomas B, Fahmy E, et al. Poverty, wealth and place in Britain, 1968 to 2005. Bristol: The Policy Press; 2007.

22. Statistics OoN. Household total wealth in Great Britain: April 2018 to March 2020 ONS; 2022.

23. Morell DWiP. Harvard Magazine. 2011.

24. Smith M, Noble. Developing the use of administrative data to study poverty. In: Bradshaw J, Sainsbury R, editors. Researching Poverty. Aldershot: Ashgate; 2000. p. 77-97.

25. Baker W, Starling G, Gordon D. Predicting Fuel Poverty at the Local Level. Bristol: Centre for sustainable energy; 2003.

26. Initiative OPHD. Alkire-Foster Method: OPHI’s method for multidimensional measurement Oxford: OPHI: University of Oxford; 2022 [Available from: <https://ophi.org.uk/research/multidimensional-poverty/alkire-foster-method/>.

27. Alkire S, Kanagaratnam U. Revisions of the global multidimensional poverty index: indicator options and their empirical assessment. Oxford Development Studies 2021;49(2):169-83.

28. Government S. Scottish Index of Multiple Deprivation 2020 Edinburgh: Scottish Government; 2020 [Available from: <https://www.gov.scot/collections/scottish-index-of-multiple-deprivation-2020/?utm_source=redirect&utm_medium=shorturl&utm_campaign=simd>

29. McLennan D, Noble S, Noble M, Plunket E, Wright G, Gutacker N. The English Indices of Multiple Deprivation. London: Ministry of Housing, communities and local government; 2019.

30. Gordon D. Census Based Deprivation Indices: Their Weighting and Validation. Journal of Epidemiology and Community Health. 1995;49:S39-S44.

31. Munro J. Munroe, Jack. 2022. We’re pricing the poor out of food in the UK – that’s why I’m launching my own price index. The Guardian. (<https://www.theguardian.com/society/2022/jan/22/were-pricing-the-poor-out-of-food-in-the-uk-thats-why-im-launching-my-own-price-index> ) The Guardian. 2022.

32. Townsend P. Poverty in the United Kingdom. Harmondsworth: Penguin; 1979.

33. McAuley C, Hillyard P, McLaughlin E, Tomlinson M, Kelly G, Patsios D. The Necessities of Life in Northern Ireland. Belfast: Queen’s University Belfast; 2003.

34. Nandy S, Main G. The Consensual Approach to Child Poverty Measurement. Bergen: Comparative Research Programme on Poverty; 2015.

35. Treanor M. Deprived or not deprived? Comparing the measured extent of material deprivation using the UK government’s and the Poverty and Social Exclusion surveys’ method of calculating material deprivation. Qual Quant. 2014;48:1337–46.

36. Main G, Bradshaw J. Child poverty and social exclusion: Final report of 2012 PSE study. PSE; 2014.

37. Main G, Bradshaw JR. Children's necessities : trends over time in perceptions and ownership. The Journal of Poverty and Social Justice. 2014:193-208.

38. Davey Smith G, Gordon D, Kelly M, Nandy S, Subramanian S. Inequalities in health in India: the methodological construction of indices and measures. UK department for international development; 2003.

39. Davis A, Hirsch D, Padley M, Marshall L. How much is enough? Research consensus on minimum household needs. Loughborough: Centre for research in social policy, Loughborough University; 2015.

40. Fahmy E, Gordon D. Mapping deprivation in the South West. Bristol: Townsend centre for international poverty research, University of Bristol; 2002.

41. Davis A, Stone J, Blackwell C, Padley M, Shepherd C, Hirsch D. A minimum income standard for the United Kingdom in 2022. York: Joseph Rowntree Foundation; 2022.

42. Treanor M. FALLING THROUGH THE CRACKS:

THE COST OF THE SCHOOL DAY FOR FAMILIES LIVING IN IN-WORK AND OUT-OF-WORK POVERTY. Scottish Affairs. 2018;27(4):486-511.

43. Naven L, Egan J, Sosu EM, Spencer S. The influence of poverty on children’s school experiences: pupils’ perspectives. Journal of Poverty and Social Justice. 2019;27(3):313-31.

44. Hirsh D, Lee T. The Cost of a Child in 2021. London: Child Poverty Action Group; 2021.

45. Kyprianou P. Getting By? A year in the life of 30 working families in Liverpool. 2015.

46. Foundation F. The broken plate: the state of the nation’s food system London: Food Foundation; 2021.

47. Salway S. Long-term ill-health, poverty and ethnicity. York: Joseph Rowntree Foundation; 2007.

48. Kempson E. Life on a Low Income. York: Joseph Rowntree Foundation; 1996.

49. McKendrick. JH, Brown. Listening to the lived experience of poverty in Scotland: An evidence review of issues pertaining to income, expenditure and wealth. SPIRU; 2018.

50. commission Sm. Measuring Poverty 2020. London: SMC The Legatum Institute; 2020.

51. commission Sm. Measuring Poverty before the Covid-19 pandemic. London: SMC The Legatum Institute; 2021.

52. Gordon D, Lloyd L, Heslop P. Jersey health survey. Bristol: University of Bristol; 2001.

53. Lister R, Beresford, Peter, Green, David and Woodward, Kirsty. Where are the poor in the future of development research? In: Bradshaw JaS, Roy editor. Researching Poverty. Aldershot.: Ashgate; 2000. p. 77-97.

54. Flaherty J. Getting by, Getting Heard: Poverty and Social Exclusion in the Borders: Listening to the Voices of Experience. Glasgow: Poverty Alliance; 2008.

55. Pemberton S, Sutton E, Fahmy E. A Review of the Qualitative Evidence Relating to the Experience of Poverty and Social Exclusion. Bristol: University of Bristol; 2013.

56. Green S, Hickman P. Residents’ Stories from Six Challenging Neighbourhoods. Centre for Regional Economic and Social Research Sheffield Hallam University; 2010.

57. Batty E, Cole I. Resilience and the Recession in Six Deprived Communities: Preparing for Worse to Come? York: Joseph Rowntree Foundation; 2010.

58. Dean H, Shah A. Insecure families and low-paying labour markets: Comments on the British experience. Journal of Social Policy. 2002;31(1):61-80.

59. Bradshaw J, Noble M, Bloor K, Huby M, McLennan D, Rhodes D, et al. A Child Well-Being Index at Small Area Level in England. Child Indicators Research. 2009;2:201-19.

60. Dyson A, Hertzman C, Roberts H, Tunstill J, Vaghri Z. Childhood Development, Education and Health Inequalities. London: Institute of Health Equity; 2010.

61. Maguire S, McKay E. Young, Female and Forgotten. Young Women’s Trust; 2017.

62. Garmezy N. Resilience and vulnerability to adverse developmental outcomes associated with poverty. American Behavioral Scientist. 1991;34:416-30.

63. Reay D. Mostly Roughs and Toughs: Social Class, Race and Representation in Inner City Schooling. Sociology. (2004). ;38(5):999-1017.

64. Reay D. Beyond Consciousness?: The Psychic Landscape of Social Class. Sociology. 2005;39(5):911-28.

65. Cohen R, Coxall J, Craig G, Sadiq-Sangster A. Hardship Britain, Being poor in the 1990s. London: CPAG; 1992.

66. Barnes H, Garratt E, McLennan D, Noble M. Understanding the Worklessness Dynamics and Characteristics of Deprived Areas. London: Department for Work and Pensions; 2011.

67. Rice B. Against the odds: An investigation comparing the lives of children on either side of Britain’s housing divide. London: Shelter; 2006.

68. Ridge T. Living with poverty: A review of the literature on children’s and families’ experiences of poverty. Centre for the Analysis of Social Policy, University of Bath: Department for Work and Pensions; 2009.

69. United Nations Department of Economic and Social Affairs. Rethinking Poverty: Report on the World Social Situation 2010. New York: United Nations; 2009.

70. Ghate D, Hazel N. Parenting in poor environments: stress, support and coping. London: Jessica Kingsley Publishers; 2002.

71. Crisp R, Robinson D. Family, Friends and Neighbours: Social Relations and Support in Six Low Income Neighbourhoods. Sheffield: Centre for Regional and Economic Research, Sheffield Hallam University; 2010.

72. Gillies V. Childrearing, class and the new politics of parenting. Sociology Compass. 2008;2(3):1079-95.

73. Walker R. The Shame of Poverty. Oxford: Oxford Academic; 2014.

74. Charlesworth SJ. A phenomenology of working-class experience. Cambridge: Cambridge University Press; 2000.

75. McGarvey D. Poverty Safari: Understanding the anger of Britain's underclass. Hampshire: Pan Macmillan; 2018.

76. Athwal B, Brill L, Chesters G, Quiggin M. Recession, Poverty and Sustainable Livelihoods in Bradford. York: Joseph Rowntree Foundation; 2011.

77. Brown GW, Moran PM. Single mothers, poverty and depression. Psychological Medicine. 1997;27:21-33.

78. Parker S, Pharaoh R. Just Coping: A new perspective on low-income families. Maidstone: Kent County Council; 2008.

79. Crowley A, Vulliamy C. Listen up!: children and young people talk about poverty. Save the Children UK 2007.

80. Wilkinson RG, Pickett KE. The Spirit Level: Why More Equal Societies Almost Always Do Better. London: Allen Lane; 2009.

81. Lister R. ‘To Count for Nothing’: Poverty Beyond the Statistics. Journal of the British Academy. 2015;3:139-65.

82. Sen A. Poor, relatively speaking. Oxford Economic Papers. 1983;35:153-67.

83. Sayer A. Class, Moral Worth and Recognition. Sociology. 2005;39(5,):947-63.

84. Charlesworth SJ, Gilfillan P, Wilkinson R. Living Inferiority. British Medical Bulletin. 2004;69:49-60.

85. Scheff TJ. Shame and the Social Bond: A Sociological Theory. Sociological Theory. 2000;18(1):84–99.

86. Tyler I. Stigma: The machinery of inequality. London: Zed books; 2020.

87. Skeggs B. Classifying Practices: Representations, Capitals and Recognitions In: Mahoney P, Zmroczek C, editors. Class Matters: "Working Class" Women's Perspectives on Social Class: Taylor and Francis; 2005. p. 127-42.

88. Gosling VK. I've always managed, that's what we do': Social capital and women's experiences of social exclusion. Sociological Research Online. 2008;13(1):1-18.

89. Sutton E, Pemberton S, Fahmy E, Tamiya Y. Stigma, shame and the experience of poverty in Japan and the United Kingdom. Social Policy and Society. 2014;13(1):143-54.

90. Walkerdine V. Class in the Consulting Room. Psychotherapy and Politics International 2007;5(1):23–8.

91. Baillie R. An examination of the public discourse on benefit claimants in the media. Journal of Poverty and Social Justice. 2011;19(1):67-70.

92. Patrick R. Living with and responding to the ‘scrounger’ narrative in the UK: exploring everyday strategies of acceptance, resistance and deflectio. Journal of Poverty and Social Justice. 2016;24(3):245-59.

93. Wincup E, Monaghan M. Scrounger narratives and dependent drug users: welfare, workfare and warfare. Journal of Poverty and Social Justice, . 2016;24(3):261-75.

94. Featherstone M. Being-in-Hull, Being-on-Bransholme: Socio-economic decline, regeneration and working-class experience on a perio-urban council estate. City. 2013;17(2):179-96.

95. Peacock M, Bissell P, Owen J. Dependency denied: Health inequalities in the neo-liberal era. Social Science & Medicine Volume. 2014;118:173-80.

96. Wilkinson R, Pickett K. The inner level: How more equal societies reduce stress, restore sanity and improve everyone’s wellbeing. UK: Allen Lane; 2018.

97. A. C. Stigmatization and public health ethics. Bioethics. 2013;27(2):74-80.

98. Richeson J.A., J.N. S. A Social Psychological Perspective on the Stigmatization of Older Adults In: L.L. C, C.R. H, editors. When I'm 64. Washington (DC): National Academies Press (US); 2006.

99. WH. VB. Measuring health-related stigma - a literature review. Psychology Health Medicine. 2006;11(3):307-34.

100. Solanke I. The stigma of being Black in Britain. Identities. 2018;25(1):49-54.

101. Skeggs B. (Dis)identifications of class: On not being working class. London: SAGE Publications Ltd; 2002.

102. Harris J, Treanor M, Sharma N. Below the breadline: A year in the life of families in poverty. Ilford: Barnardo’s; 2009.

103. Millar J, T. R. No margin for error: fifteen years in the working lives of lone mothers and their children. Journal of Social Policy. 2018:1-17.

104. Harrison E. Bouncing back? Recession, resilience and everyday lives. Critical Social Policy. 2013;33: 97-113.

105. McKendrick JH, Cunningham-Burley S, Backett-Milburn K. Life in Low Income Families in Scotland: Research Report. Edinburgh: Scottish Executive Social Research; 2003.

106. Kempson E, McKay S, Willitts M. Characteristics of Families in Debt and the Nature of Indebtedness. Leeds: DWP; 2004.

107. Kempson E, Whyley C. Kept Out or Opted Out? Understanding Financial Exclusion. Bristol: The Policy Press; 1999.

108. Reay D, Crozier G, Clayton J. Strangers in Paradise? Working-class students in elite universities. Sociology, . 2009;43(6):1103-21.

109. Chase E, Walker R. The co-construction of shame in the context of poverty: Beyond a threat to the social bond. Sociology. 2012;published online 17 October 2012.

110. Batty E, Flint J. Self-Esteem, Comparative Poverty and Neighbourhoods. York: Joseph Rowntree Foundation; 2010.

111. Walkerdine V. Communal Beingness and Affect: An Exploration of Trauma in an Ex-industrial Community. Body & Society. 2010;16(1):91-116.

112. Collective FE. Foundational Economy: the infrastructure of everyday life. Manchester: Manchester University Press; 2018.

113. Esping-Andersen G. The three worlds of welfare capitalism New Jersey: Princeton University Press; 1990.

114. Gough I. Move the debate from Universal Basic Income to Universal Basic Services UNESCO Inclusive Policy Lab; 2021.

115. Nations U. About extreme poverty and human rights: Special Rapporteur on extreme poverty and human rights Geneva: Office of the United Nations High Commissioner for Human Rights (OHCHR); 2012 [Available from: <https://www.ohchr.org/en/special-procedures/sr-poverty/about-extreme-poverty-and-human-rights>.

116. Bray R, de Laat M, Godinot X, Ugarte A, Walker R. Realising poverty in all its dimensions: A six-country participatory study. World Development 2020;134:1-10.

117. Broady R, Button A, Campbell S, Chase E, Corlyon J, Currie A, et al. Understanding Poverty in All its Forms: A participatory research study into poverty in the UK. London: ATD Fourth World; 2020.
