# Supplementary material for: What is missing from how we measure and understand the experience of poverty and deprivation in population health analyses?
Source: Eur J Public Health. 2023 Oct 20;33(6):974–80. doi: 10.1093/eurpub/ckad174 (PMC10710332; doi:10.1093/eurpub/ckad174)
Supplement: ckad174_Supplementary_Data [file ckad174_supplementary_data.zip › ckad174_Supplementary_Data/ejph-2023-04-om-0206-File007.docx]

**Appendix 2 – Concept definitions table**

| **Concept** | **Section** | **Description** |
| --- | --- | --- |
| Ability to make plans | Outcomes of psychological effects | Having the resources (time, money, energy) to be able to plan ahead, which allows you to increase your resilience. |
| Emotional labour, effort and support | Outcomes of psychological effects | The energy expended on coping emotionally. How much of this effort you (are expected to) expend, for your own and for others' coping, and how much others (are willing to) expend these efforts to help you cope. This may be seen as emotional resilience, which is dependent on resources; being there for others is only possible when you yourself are secure. |
| Masking poverty | Outcomes of psychological effects | The work undertaken to hide conditions of poverty from family or the wider community, generally in order to limit the impact of stigma, which comes with costs - both financial and mental/emotional. |
| Access to education | Class, work and education | Equity and access to good quality education at each level. |
| Class Processes | Class, work and education | Class-delineating process, whereby people learn how to act in certain contexts to differentiate themselves from other classes and so make use of power systems which will grant them opportunities and block those opportunities for others. Including: discrimination; habitus and distinction; opportunity hoarding; social closure. |
| Exclusion from education and work | Class, work and education | Both formal and informal, including self-exclusion. The ways in which you become cut off from education and/or work. This often begins in education, with enforced exclusion or informal non-attendance. |
| Exploitation and domination | Class, work and education | The process by which paid and unpaid work create uneven power relations. |
| Formal recognition and protection of minority status | Class, work and education | Whether or not a characteristic is recognised as protected has implications for how disadvantages can be mitigated. |
| Identity based on activity | Class, work and education | The sense of self which activities such as work and study can bring. |
| (Intersecting) personal characteristics | Class, work and education | Although characteristics are often considered separately, they do not exist that way in reality. Identities intersect multiple characteristics that, based on the underlying power dynamics of a society, confer a range of advantages and disadvantages, which individuals must navigate. |
| Learning and training | Class, work and education | From pre-school to tertiary education, and including training taken as part of work. |
|  |  |  |
| Protection and organisation | Class, work and education | The structures within educational and workplace settings that students and employees can use to organise and thereby to protect their rights. Including, but not limited to trade union membership. |
| Punishment | Class, work and education | The severity of the punishments administered when you break or transgresses social rules. This may involve monetary costs, or costs in time, or exclusion from society - which can be temporary or permanent, including imprisonment. Measurements can consider frequency and severity, as well as looking at how certain social groups are affected more or less than others. |
| Qualifications and attainment | Class, work and education | The skills learned in education that grant access to further education and to employment. |
| Quality of management or teaching | Class, work and education | Often measured by outputs, but can be more qualitatively understood. Whether or not you are supported by those directly responsible for you in education or work. Including bullying and mis-use of power, where hierarchical power dynamics are used against you, causing physical or psychological harm. |
| Quality of work and education | Class, work and education | Measures of work quality look at hours worked, control over work tasks, time and environment, ability to progress and other elements. Measures of educational quality look at attainment, satisfaction. |
| Social Dislocation | Class, work and education | The experience of moving from one class or group to another. |
| Stigma | Class, work and education | Negative social judgement based on perceived characteristics. |
| Structures of services | Class, work and education | Societal services, including education, health, transport, legal services, social security, criminal justice and policing.  How free-to-access services interact with paid-for services, and how these structures determine access and quality, and feed into class processes. |
| Time pressures | Class, work and education | The control people have over their time and what they need to fit into that time. Work life balance (WLB) is a part of this, but is also a particularly classed concept - with different groups of people having different WLB expectations as well as being differently able to achieve WLB. |
| Work | Class, work and education | Both paid and unpaid work – how accessible it is, what people receive in compensation for it, and the legal and social rules surrounding work and working conditions. |
| Anxiety | Internal(ised) feelings | A feeling of fear or dread, can be in response to external situations or the result of a mental health condition, or both. This feeling may cut people off from support, depending on how that support is offered and accessed (e.g. if a healthcare call comes from an unknown number, someone may be too anxious to pick up).  When fear is normalized (e.g. during the pandemic), people do not know when and where to seek help with anxiety. |
| Defensiveness and Anger | Internal(ised) feelings | Aggressive or deflective feelings used to cope with shame, stigma or fear. |
| Humiliation or pride | Internal(ised) feelings | Sense of deep embarrassment which can lead to depressive feelings, and the opposing feeling of self-worth that can contribute to happiness. |
| Powerlessness or agency | Internal(ised) feelings | The opposing feelings of lacking power and of being able to take action, which shape experiences and identity. |
| Meaningfulness or boredom | Internal(ised) feelings | An element of work (paid or unpaid), a sense of purpose which allows people to build identities around what they do, and the opposite of that feeling. |
| Security | Internal(ised) feelings | Physical: neighbourhood safety from busy traffic, pollution, violence. Financial: security of income, of work or social protection, of wealth.  Social: security as a member of a community, security of friendships. Felt: How safe you feel in each/any of these areas, as well as how safe you actually are.   How likely positive impacts are to endure, or how threatened they may be. |
| Sense of lost potential | Internal(ised) feelings | The feeling that you have been unable to fulfil your potential - that you were capable of more than circumstances gave you the opportunity to achieve. This can be felt in reference to a particular situation - for example on a single piece of work - or more generally over time, and refers to the lost potential of others in your family or community, and to the general loss to all when any individual is unable to fully contribute their skills and abilities. |
| Shame | Internal(ised) feelings | Internalised judgement. Encompasses feelings of failure, guilt and inferiority and the sense of being a burden on others. |
| Stress | Internal(ised) feelings | Mental or emotional strain, felt as a result of adverse circumstances. |
| Barriers to access services | Power structures | Where services are provided, whether there are barriers in place limiting access. This can be because of intentional rationing or poor design. Built in costs, time commitments, difficult application processes or social stigmas can prevent or discourage service use. |
| Democracy | Power structures | One structure in which individuals have the opportunity to influence power and decision-making. |
| Governance | Power structures | How power and policies are negotiated and created. |
| Governments and policy | Power structures | Who has political power and what their priorities are. |
| How power is experienced | Power structures | Including: the ability to challenge those in power (both legally and emotionally being able to); being able to see yourself (and those like you) in (various positions of) power; membership of groups and organisations; political agency and voice; power distribution; and the varieties of participation (both top-down and bottom up) that are open to you. |
| Legal rules | Power structures | Including how power structures become formalised into legal rules. |
| Polities and institutions | Power structures | Large scale groups which impact how policy is created and enacted and how people identify themselves and others. |
| Power structures | Power structures | The large-scale composition of power, which is built and maintained over generations, which impacts and is impacted upon by other power processes. Includes cultures, ecology, history and the relationships between societies. |
| Public policy | Power structures | The policies which shape the context in which societies function and individuals live. |
| Rights and control | Power structures | Knowing and being able to access your rights in various situations, and the control over your circumstances that your access to these rights can give you.  For example, in housing, the varying levels of control people have over where they live, how long they can stay there, when they can leave or move, what rules they can enforce on those around them and what rules they need to follow themselves, which are based on tenure types, rights, locality and personal interactions. |
| Being (in)visible | How others see you | The most extreme form of the feeling which can come from social judgement – the sense of lacking societal value to the extent that you feel you have no social identity, or that your community cannot recognise your identity. Conversely, the feeling that you are under constant scrutiny and have no privacy, for example when you are part of a small community where everyone is aware of your financial circumstances. |
| Being Valued | How others see you | The sense of being judged by others and the sense of being valued, or respected, as someone who matters to society. This can be affected by the experience of poverty, but also by other experiences and characteristics - e.g. age, ethnicity, gender, disability.  Value is often experienced in terms of money - how much money is spent on you (e.g. within a workplace) can indicate how valued you are - but can also be about time - how much of someone's (e.g. a GP's) time you are given. |
| Belonging | How others see you | Whether or not others see you as part of a community, which impacts your own sense of belonging in a place or in a social or identity group. |
| Collective imagination | How others see you | A shared understanding of reality, based on shared ideas and/or attributes, which allows groups, communities or societies to progress and function collectively. Can have both positive and negative impacts. |
| Fairness or justice | How others see you | The sense that systems of power and distribution work in a just way, that people in general deserve what they have - and who decides what is considered 'fair' or 'just'. Can also be thought of as accountability.  The opposite of this is a sense of abandonment or helplessness. |
| Being unable to participate | Costs and income | Activities, services and goods that are not accessed, usually as a result of the interaction between costs and income, although non-material barriers also exist. |
| Costs | Costs and income | How much income or wealth you need to purchase items and services.  E.g.: childcare, housing, internet, transport, healthy diet, disability, fuel, pregnancy, parenthood, leisure, days out, holidays, special occasions, celebrations, clothing, equipment, toys, haircuts. |
| Debt | Costs and income | Debt can allow people to avoid the immediate impacts of going without a necessary cost. There are various types of debt, and the poorest tend to be exposed to the most harmful. |
| Income based measures | Costs and income | Ways of measuring poverty that focus primarily on income. Income can be derived from paid employment, from social security, from wealth and assets. Including: depth of poverty; income distribution – both over society and within households; and periodicity of poverty. |
| Poverty premium | Costs and income | The phenomenon by which those living in poverty pay more for the same items or services due to e.g., different forms of billing, lower costs if you can pay upfront, savings if you can afford insurance, the (in)ability to buy good quality goods which will last, access to affordable credit. |
| Progressiveness of taxation | Costs and income | How taxation is used to influence the distribution of wealth - whether that further benefits those in positions of power or aims to increase equality. |
| Wealth and assets | Costs and income | The stock of income and resources held, such as savings of money, housing ownership, etc. |
| Homelessness | Housing and neighbourhood | Not having appropriate/adequate housing that meets your housing needs. Both the extent of homelessness (and homelessness risk) and how harmful it is – how long it lasts, how much stress and anxiety it causes and what supports are in place for people experiencing it. |
| Housing Quality | Housing and neighbourhood | Includes affordability (particularly important in areas with high levels of tourism), size/overcrowding, damp, warmth (cost and type of fuel), security, furniture, internet access, decor, the amount that you need to move house, suitability (for disabled people or people with a mental illness), choice of area to live in. Tenure - which can be about power within a neighbourhood as well as security of housing. |
| Neighbourhood | Housing and neighbourhood | Attributes of the area in which you live, including: accessibility of amenities, such as healthcare, education, cultural, city centre; the physical environment – both built and natural and how decisions are made over that environment; remoteness; tensions within communities, which can be based on class, housing tenure or illness and experienced as ‘intolerance’ or ‘us and them mindsets’; transport accessibility, affordability, timeliness and safety. |
| Social connections | Housing and neighbourhood | The social supports, responsibilities and tensions that exist within your most immediate social contacts - households, families and friendships - and the material or emotional resources these can grant access to. |
